# A Novel Zero-Velocity Interval Detection Algorithm for a Pedestrian Navigation System with Foot-Mounted Inertial Sensors

**DOI:** 10.3390/s24030838

**Published:** 2024-01-27

**Authors:** Xiaotao Wang, Jiacheng Li, Guangfei Xu, Xingyu Wang

**Affiliations:** College of Astronautics, Nanjing University of Aeronautics and Astronautics, Nanjing 211100, China; wangxtao1977@nuaa.edu.cn (X.W.);

**Keywords:** pedestrian navigation, adaptive thresholding, zero-velocity interval detection, gait frequency analysis, ZUPT algorithm

## Abstract

The zero-velocity update (ZUPT) algorithm is a pivotal advancement in pedestrian navigation accuracy, utilizing foot-mounted inertial sensors. Its key issue hinges on accurately identifying periods of zero-velocity during human movement. This paper introduces an innovative adaptive sliding window technique, leveraging the Fourier Transform to precisely isolate the pedestrian’s gait frequency from spectral data. Building on this, the algorithm adaptively adjusts the zero-velocity detection threshold in accordance with the identified gait frequency. This adaptation significantly refines the accuracy in detecting zero-velocity intervals. Experimental evaluations reveal that this method outperforms traditional fixed-threshold approaches by enhancing precision and minimizing false positives. Experiments on single-step estimation show the adaptability of the algorithm to motion states such as slow, fast, and running. Additionally, the paper demonstrates pedestrian trajectory localization experiments under a variety of walking conditions. These tests confirm that the proposed method substantially improves the performance of the ZUPT algorithm, highlighting its potential for pedestrian navigation systems.

## 1. Introduction

As the need for improved pedestrian mobility continues to grow, the focus of research has increasingly shifted toward innovative pedestrian navigation solutions. Real-time position, as facilitated by pedestrian navigation, can boost mission effectiveness and success probability in emergency rescue and single-soldier operations. Navigational precision is particularly crucial in complex indoor environments, underground parking lots, and large theaters, among others.

Presently, pedestrian navigation technology falls into three primary categories: those based on Global Navigation Satellite System (GNSS) positioning technology, those relying on pre-installed devices, and those utilizing self-contained sensors, while GNSS-based positioning technology is highly developed and widely applicable, it is difficult to maintain positioning accuracy indoors or in areas where GNSS signals are unavailable. The second approach uses signals like Ultra-Wideband (UWB) [1], Bluetooth [2], Long-Term Evolution [3], and Wi-Fi [4] to determine location. Although the accuracy is high, this method depends on the pre-installed signal transmission equipment, which can increase costs. In contrast, self-contained sensor-based positioning techniques predominantly utilize accelerometers, gyroscopes, and magnetometers to perceive pedestrian location information. This method is cost-effective, highly independent, requires no pre-installation, and is less susceptible to interference. Moreover, advancements in microelectronics have considerably improved the size and precision of these sensors, making this approach much more convenient to implement.

However, low-cost inertial measurement units (IMUs) suffer from high noise levels and poor stability over the short and long terms. These errors continuously accumulate over the course of inertial navigation [5]. Without effective noise suppression methods, these accumulated errors can rapidly become substantial, often exceeding one meter within seconds of the navigation time for consumer-grade IMUs [6]. The zero-velocity update (ZUPT) method presents a mature solution to this issue. By segmenting a pedestrian’s gait into stance and swing phases, the ZUPT method effectively reduces the inertial drift within the stance phase [7]. A pedestrian’s gait exhibits cyclical variations corresponding to the alternating movement of the legs. At specific points, one foot makes full ground contact, reducing its velocity to nearly zero, thus creating a zero-velocity interval. This interval can be exploited to correct errors and reduce the impact of error accumulation. Accurate extraction of these zero-velocity intervals is therefore crucial for the effectiveness of this algorithm. Numerous researchers have proposed a variety of methods for detecting these intervals.

Tian et al. introduced a zero-velocity detection algorithm employing multiple threshold constraints [8], while Wagstaff et al. developed an adaptive-threshold adjustment method based on recognizing a pedestrian’s motion states through inertial sensors [9]. Ma et al. proposed a technique using high-resolution pressure sensors to detect zero-velocity intervals [10]. Another type of method uses machine learning to process and classify accelerometer and gyroscope data to detect zero-velocity intervals. Common techniques include Support Vector Machines (SVMs), Random Forests [11], Convolutional Neural Networks (CNNs) [12], and Long Short-Term Memory (LSTM) [13]. For example, Shi et al. suggested a thresholdless gait sequence segmentation algorithm using LSTM networks to detect zero-velocity intervals [14]. However, these methods typically require extensive training data, which can be time-consuming.

**Figure 1:**
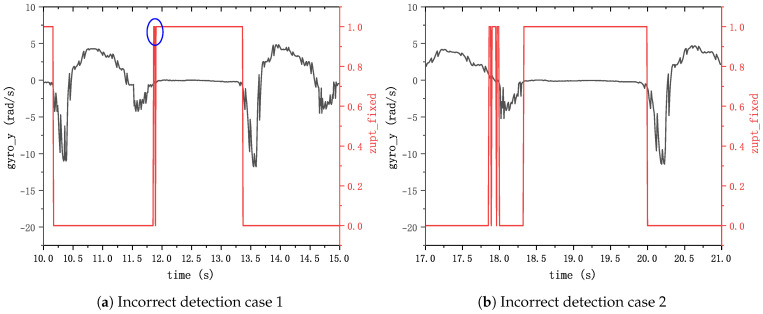
Incorrect detection examples when the threshold does not match the pedestrian’s speed.

Conversely, threshold-based detection methods often use fixed thresholds. While they are effective in identifying zero-velocity intervals during consistent pedestrian motion, the randomness of pedestrian movement can result in false positives and negatives, especially when the detection threshold does not fit the pedestrian walking speed [15]. The most common incorrect detection examples are shown in Figure 1. The length of the zero-velocity interval will adversely affect the pedestrian motion solution, especially the distance moved.

Many researchers have turned to time–frequency analysis methods to extract pedestrian gait frequencies and adaptively adjust the thresholds accordingly. For instance, Sun et al. introduced a method that involves auto-correlation of acceleration data followed by Fast Fourier Transform (FFT)-based gait frequency extraction [16]. Similarly, Tian et al. proposed a zero-velocity detection method using the Smoothed Pseudo WVD (SPWVD) to extract frequency domain information from gyroscope data, eliminating harmonics to obtain the gait frequency [17]. These techniques use time–frequency analysis to analyze pedestrian gait from inertial sensor data, effectively capturing gait frequency during movement. However, common time–frequency analysis methods, such as Short-Time Fourier Transform (STFT) [18] and the Wigner–Ville Distribution (WVD) [19], have their limitations. STFT uses a fixed window, leading to a trade-off between frequency and time resolution, resulting in lower computational accuracy [20,21]. It is difficult for the WVD to suppress crossover terms [22]. The Smoothed Pseudo Wigner–Ville Distribution (SPWVD), an improvement over the WVD, introduces window functions in both the time and frequency domains, effectively mitigating cross-term interference but at the cost of increased computational complexity.

To overcome these limitations, this paper introduces the adaptive window gait frequency-based zero-velocity detector (AWGF-ZVD). Compared with the STFT, this method adaptively adjusts the window size to better accommodate the variations in gait frequency from inertial data, thus making the algorithm more sensitive to changes in the pedestrian gait frequency. At the same time, it does not have the cross-talk problem that exists in the WVD and is simpler than the SPWVD. Compared with the fixed-threshold method, it can adapt to the changes in pedestrian gait, which improves the correct detection rate. It does not require a lot of data like machine learning methods do.

The remainder of this paper is structured as follows: Section 2 analyzes the characteristics of pedestrian gait and the frequency spectral lines; Section 3 introduces the AWGF-ZVD method and the architecture of pedestrian navigation algorithms; Section 4 presents comparative experiments on various zero-velocity detection algorithms, single-step estimation experiments, and pedestrian trajectory localization experiments to assess the proposed algorithm’s efficacy; and Section 5 summarizes the research findings.

## 2. Analysis

For foot-mounted inertial navigation, the phenomenon of cumulative errors caused by continuous motion is commonly observed. Therefore, dividing the human gait into two phases—the static state and the motion state—and applying corresponding error compensation methods has been proven to be an effective and feasible strategy.

### 2.1. Analysis of Gait Characteristics

**Figure 2:**
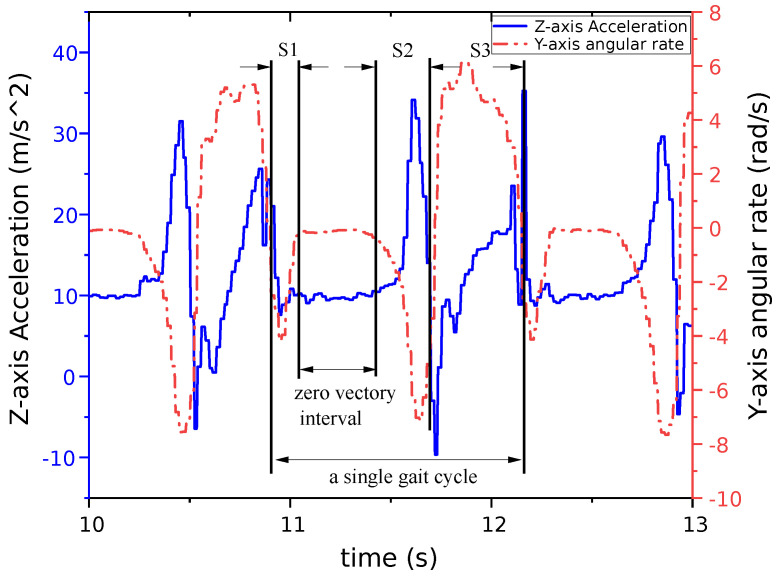
Outputs of the *z*-axis accelerometer and *y*-axis gyroscope in the gait cycle. The solid blue line shows the *z*-axis accelerometer data, and the dashed red line represents the *y*-axis gyroscope data.

Figure 2 presents the outputs of the *y*-axis gyroscope and *z*-axis accelerometer during a complete gait cycle under stable walking conditions in the experimental setup described above. A full gait cycle is divided into four segments: S1, zero-velocity interval, S2, and S3. For the foot equipped with inertial sensors, S1 represents the phase from the heel strike to full foot–ground contact. During this phase, the foot rotates around the *y*-axis, resulting in a negative output from the *y*-axis gyroscope. This is followed by a phase of full foot–ground contact, known as the zero-velocity interval. In this interval, the sensor output is nearly constant, indicated by the *y*-axis angular velocity being close to zero and the *z*-axis acceleration approximating gravitational acceleration. The S2 phase encompasses the period from heel lift-off to the foot leaving the ground, during which the *y*-axis gyroscope output remains negative. Finally, the S3 phase involves thigh swinging and forward body movement until the completion of the cycle, with the *y*-axis angular velocity being positive during this stage.

### 2.2. Analysis of the Fourier Spectrum

**Figure 3:**
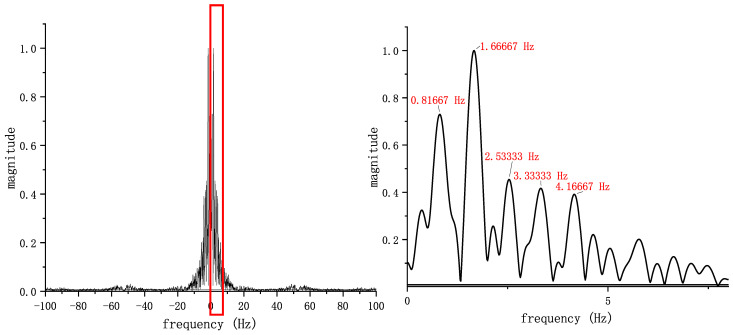
The Fourier spectral lines of the pedestrian gait.

According to the preceding analysis, the *y*-axis gyroscope output can be a fair depiction of the periodic variation in the human gait. As a result, a time–frequency analysis method may be used to derive the gait frequency of the human body. Initially, several sets of data are randomly extracted from the pedestrian’s stable walking process, leading to the determination that the average walking frequency of a pedestrian is approximately 0.8 Hz. A Fourier Transform spectrum is then extracted from a 3 s output of the *y*-axis gyroscope data during stable walking. As demonstrated in Figure 3, the amplitude is primarily concentrated within the 0–10 Hz range. Upon magnifying this segment, the spectrum reveals several peaks, with the highest peak corresponding to a frequency of 1.67 Hz, approximately twice the human gait frequency $2f_{0}$ (0.82 Hz). The other peaks at frequencies of 2.53 Hz, 3.33 Hz, and 4.17 Hz correspondingly align with $3f_{0}$, $4f_{0}$, $5f_{0}$, respectively, indicating their harmonic relationships with the fundamental gait frequency.

Therefore, the key challenge lies in extracting effective frequencies among the several peaks. Hence, this paper proposes an adaptive window-based time–frequency analysis method, which dynamically adjusts the window parameters in response to changes in the frequency domain information.

## 3. Methods

**Figure 4:**
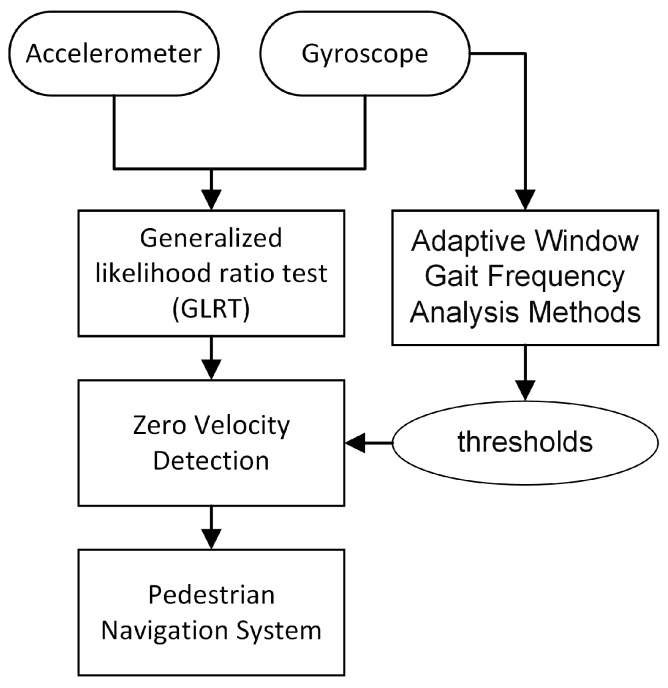
The algorithm workflow.

The method’s procedural workflow is depicted in Figure 4. Initially, the inertial measurement unit (IMU) collects data followed by the correction of biases in the data. Subsequently, a zero-velocity detection algorithm is applied to extract zero-velocity intervals from the pedestrian gait. Next, an Extended Kalman Filter (EKF) based on quaternions is used to obtain attitude information. Then, through compensation for zero-velocity errors and integral calculations, the final position and velocity information is determined. This method can improve the positioning accuracy of foot-mounted inertial navigation systems.

Unlike the current widely used data-driven methods [23,24] that often require extensive datasets for training, our method demonstrates efficacy even with smaller datasets. This not only reduces the dependence on vast data resources, but also makes our approach more accessible to a broader range of researchers with varying computational capacities.

Furthermore, a distinctive feature of our proposed algorithm is its low computational resource requirements. Unlike data-driven methods that demand significant computing power, our algorithm is designed to operate efficiently even on modest hardware configurations. This characteristic not only reduces the barrier to entry for users with limited computational resources but also makes our algorithms cost-effective solutions for a variety of applications.

### 3.1. The Adaptive Window Gait Frequency-Based Zero-Velocity Detector

The characteristics analyzed in Section 2.1 can be leveraged to design a zero-velocity detection algorithm. The zero-velocity detection algorithm proposed in this paper, based on frequency domain analysis, consists of two main components: firstly, a frequency analysis method using an adaptive window, which is employed to extract the gait frequency during pedestrian walking; secondly, a zero-velocity interval detection algorithm that adaptively adjusts its threshold based on the gait frequency.

#### 3.1.1. Adaptive Window Gait Frequency Extraction Algorithm

The Fourier Transform of a continuous-time signal x(t) can be represented as

(1) $$\begin{array}{c} \begin{array}{c} X\left(f\right)=\int_{-\infty }^{\infty }x\left(t\right)\cdot e^{-j2\pi ft}dt \end{array} \end{array}$$

where x(t) is the time domain signal, X(f) is the spectral density function of the signal, *j* is the imaginary unit, and *f* is the frequency.

However, the signal collected by the sensor is a discrete signal. After discretizing the above equation, the Discrete Fourier Transform (DFT) of the discrete signal sequence x[n] can be expressed as

(2) $$\begin{array}{c} \begin{array}{c} X\left[k\right]=\sum_{n=0}^{N-1}x\left[n\right]\cdot e^{-j2\pi kn/N} \end{array} \end{array}$$

where X[k] is the spectral density function of the discrete sequence, *N* is the sequence length, and *k* is the sequence number.

**Figure 5:**
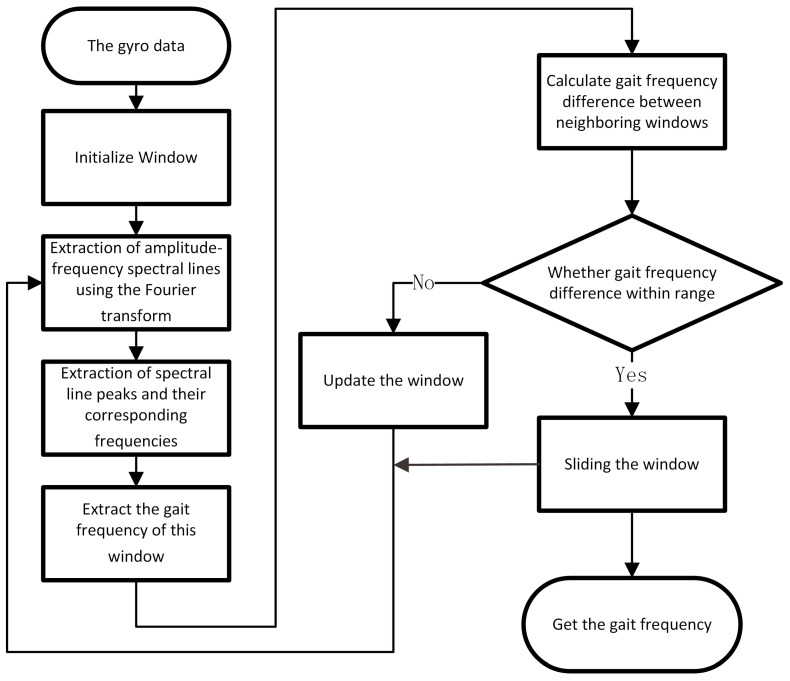
The workflow of the adaptive window gait frequency analysis method.

Building on this, the Short-Time Fourier Transform (STFT) method applies a window to the time domain signal and then performs the Discrete Fourier Transform (DFT) on each windowed segment. The method proposed in this paper involves extracting the pedestrian gait frequency within each window and then adaptively adjusting the window in response to variations in frequency to accommodate for the changes in the frequency domain. The workflow of this adaptive time–frequency analysis method is depicted in Figure 5 and described below.

1. We initialized the window for the time domain signal with an initial window size set to 600. We then extended the data at the boundaries of the window with zeros to enhance the frequency resolution. We then used DFT to extract the spectral lines.
2. For the spectral lines of the *i*-th window $W_{i}$, the adaptive multiscale-based peak detection (AMPD) algorithm [25] is applied to identify the peak frequencies $F_{p}=\left[f_{peak,1},f_{peak,2},\cdots ,f_{peak,k_{i}}\right]$ and their corresponding amplitudes $A_{p}=\left[A_{peak,1},A_{peak,2},\right.$ $\left.\cdots ,A_{peak,k_{i}}\right]$. Following this, the maximum peak of the spectrum $A_{max}$ is extracted, and the frequency associated with this peak is labeled as $f_{max}$. Its index in $F_{p}$ is noted as $index_{max}$.
3. We then find the maximum value of $A_{p}$ with an index less than $index_{max}$ and its corresponding frequency $f_{i}$. This frequency $f_{i}$ is considered as the pedestrian gait frequency $f_{human,i}$.
4. We move to the next window $W_{i+1}$ and repeat steps 2 and 3 to obtain the gait frequency $f_{human,i+1}$ of this window; we then calculate the frequency difference of the neighboring times: $\Delta f_{human,i}=f_{human,i+1}-f_{human,i}$. When the frequency difference satisfies $\Delta f_{human,i}>\gamma_{f}$, the sliding window width is reduced, and when the frequency difference satisfies $\Delta f_{human,i}<-\gamma_{f}$, the sliding window width increases. Based on the pedestrian’s average walking frequency of 0.8 Hz and maximum running frequency of 5 Hz, the frequency difference threshold $\gamma_{f}$ can be set to 0.3 Hz. Then we calculate the $f_{human,i+1}$ again.
5. Repeat steps 2–4 to acquire the pedestrian gait frequency.

#### 3.1.2. The Adaptive-Threshold Zero-Velocity Detection Method

Building upon the adaptive window frequency analysis method described in Section 3.1.1, this section reports the design of the zero-velocity detection method that utilizes an adaptive threshold based on the Generalized Likelihood Ratio Test (GLRT). The underlying principle is as follows: For the output values of the inertial sensors during walking, $\left[a_{x}\left(k\right),a_{y}\left(k\right),a_{z}\left(k\right),\omega_{x}\left(k\right),\omega_{y}\left(k\right),\omega_{z}\left(k\right)\right]$, the GLRT statistic within the sliding window can be expressed as

(3) $$\begin{array}{c} \begin{array}{c} T\left(k\right)=\frac{1}{w}\left[\frac{1}{\sigma_{a}^{2}}\sum_{j=k-\left(w-1\right)//2}^{k+\left(w-1\right)//2}{\left(a_{j}-\frac{g\cdot {\bar{a}}_{k}}{∥{\bar{a}}_{k}∥}\right)}^{2}+\frac{1}{\sigma_{\omega }^{2}}\sum_{j=k-\left(w-1\right)//2}^{k+\left(w-1\right)//2}\left[\omega_{x}^{2}\left(j\right)+\omega_{y}^{2}\left(j\right)+\omega_{z}^{2}\left(j\right)\right]\right] \end{array} \end{array}$$

where *w* denotes the size of the window, $a_{j}={\left[a_{x}\left(j\right),a_{y}\left(j\right),a_{z}\left(j\right)\right]}^{T}$, $\sigma_{a}^{2}$ and $\sigma_{\omega }^{2}$ are the variance of the acceleration noise and angular rate noise, respectively, *g* is the local gravitational acceleration $9.78\;{m/s}^{2}$, and ${\bar{a}}_{k}$ is the mean value of the acceleration at moment *k*, which can be expressed as

(4) $$\begin{array}{c} \begin{array}{c} {\bar{a}}_{k}=\frac{1}{w}\sum_{j=k-\left(w-1\right)//2}^{k+\left(w-1\right)//2}\left[\begin{array}{c} a_{x}\left(j\right)\\ a_{y}\left(j\right)\\ a_{z}\left(j\right) \end{array}\right] \end{array} \end{array}$$

By setting the GLRT threshold $\gamma_{k}$, the zero-speed detection can finally be achieved. The threshold constraint conditions are presented as follows:

(5) $$\begin{array}{c} \begin{array}{c} \left\{\begin{array}{cc} H_{1} & T\left(k\right)\leq \gamma_{k}\\ H_{0} & T\left(k\right)>\gamma_{k} \end{array}\right. \end{array} \end{array}$$

In the above formula, $H_{1}$ indicates that the interval at that moment is a zero-velocity interval, whereas $H_{0}$ signifies that the interval is not a zero-velocity interval.

In the traditional detection method, the threshold is set to a fixed value, making it only suitable for determining zero-velocity intervals during steady walking. When a pedestrian’s gait is complex and variable, the accuracy of this method is low. To address this issue, the GLRT threshold $\gamma_{k}$ is set as a dynamic value that adapts to the gait frequency, thus enhancing the robustness of the algorithm.

**Figure 6:**
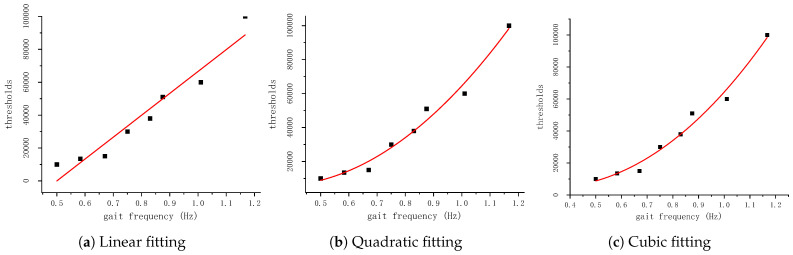
Threshold vs. gait frequency fitting curves.

Through conducting experiments and statistical analysis on the data collected at various gait frequencies, the optimal threshold for each gait condition can been determined. The relationship between gait frequency and threshold was modeled using linear, quadratic, and cubic spline interpolation, as illustrated in Figure 6.

According to Table 1, the cubic fit has a higher goodness of fit and a smaller Root Mean Square Error (RMSE); therefore, it was used as a fit for the threshold and gait frequency. The fitted curve representing the relationship between the threshold and gait frequency, as shown in Figure 6c, can be presented as $y=ax^{3}+bx^{2}+cx+d$, where *a*, *b*, *c*, and *d* are the fitting coefficients. In this paper, a=12,499.609,b=105,547.262,c=−68,955.180,d=12,312.054.

### 3.2. Pedestrian Navigation Algorithm

The IMU-based pedestrian navigation method used in this paper is divided into two parts: the adaptive zero-velocity detection algorithm and the strapdown inertial navigation algorithm. The adaptive zero-velocity detection algorithm was introduced in Section 3.1. This section will introduce the strapdown inertial navigation algorithm (SINS).

The navigation algorithm designed in this study predominantly utilizes the Kalman Filter for error correction during the zero-velocity interval, facilitating updating the attitude, velocity, and position. Consequently, the algorithm operates in a two-step process: prediction and updating. The navigation coordinate system adheres to a north–east–down (NED) orientation, aligning with the local geographical coordinate framework. Upon acquisition of IMU data, the system’s attitude is determined by updating the quaternion $q_{k}$ as

(6) $$\begin{array}{c} \begin{array}{c} q_{k}=\Omega \left(\omega_{k}dt\right)q_{k-1} \end{array} \end{array}$$

where $\Omega \left(\omega_{k}dt\right)$ is the quaternion update matrix; $\omega_{k}$ is the angular velocity at moment *k*; and Δt is the sampling interval $\Delta t=\frac{1}{f_{s}}$, where $f_{s}$ is the sampling frequency of the IMU. The position and velocity can be solved as follows:

(7) $$\begin{array}{c} \begin{array}{c} \begin{array}{cc} p_{k} & =p_{k-1}+v_{k-1}\Delta t\\ v_{k} & =v_{k-1}+\left(q_{k-1}a_{k}q_{k-1}^{*}-g\right)\Delta t \end{array} \end{array} \end{array}$$

where $p_{k}$ is the position at moment *k*; $v_{k}$ represents the velocity at moment *k*; $a_{k}$ is the acceleration at moment *k*; and $q_{k-1}a_{k}q_{k-1}^{*}$ represents a quaternion rotation, which means that the accelerometer data $a_{k}$ is rotated by the orientation represented by $q_{k-1}$, thus transforming the accelerometer data $a_{k}$ to the navigation coordinate system.

Therefore, the state update can be expressed as

(8) $$\left[\begin{array}{c} p_{k}\\ v_{k}\\ q_{k} \end{array}\right]=\left[\begin{array}{c} p_{k-1}+v_{k-1}\\ v_{k-1}+\left(q_{k-1}f_{k}q_{k-1}^{*}-g\right)dt\\ \Omega \left(\omega_{k}dt\right)q_{k-1} \end{array}\right]=\;\;\left[\begin{array}{ccc} I & Idt & O\\ O & I & \left(q_{k-1}f_{k}q_{k-1}^{*}-g\right)q_{k-1}^{*}dt\\ O & O & \Omega \left(\omega_{k}dt\right) \end{array}\right]\left[\begin{array}{c} p_{k-1}\\ v_{k-1}\\ q_{k-1} \end{array}\right]$$

where the state transition matrix is denoted as Φ,

(9) $$\begin{array}{c} \begin{array}{c} \Phi =\left[\begin{array}{ccc} I & Idt & O\\ O & I & \left(q_{k-1}f_{k}q_{k-1}^{*}-g\right)q_{k-1}^{*}dt\\ O & O & \Omega \left(\omega_{k}dt\right) \end{array}\right] \end{array} \end{array}$$

Subsequent updates can be performed using an Extended Kalman Filter in the zero-velocity interval. The error state vectors are defined as

(10) $$\begin{array}{c} \begin{array}{c} \delta x=\left[\begin{array}{ccccc} \delta P & \delta v & \delta \phi & \epsilon^{b} & \nabla^{b} \end{array}\right] \end{array} \end{array}$$

where δP is the position error vector, δv is the velocity error vector, δϕ is the attitude error vector, $\epsilon^{b}$ is the gyroscope bias error, and $\nabla^{b}$ is the accelerometer bias error vector.

The measurement equation of this system is

(11) $$\begin{array}{c} \begin{array}{c} \delta z_{k}=H\delta x_{k}+\nu_{k} \end{array} \end{array}$$

where $\delta z_{k}$ is the error measurement of time *k*, H is the measurement matrix, and $\nu_{k}$ is the measurement noise. When the ZVI is detected, the error vector δz can be obtained by performing a subtraction operation between the velocity and the heading attitude calculated by the SINS and in the ZVI. In this paper, the measurement matrix can be expressed as

(12) $$\begin{array}{c} \begin{array}{c} H=\left[\begin{array}{ccccc} 0_{3\times 3} & I_{3\times 3} & 0_{3\times 3} & 0_{3\times 3} & 0_{3\times 3}\\ 0_{1\times 3} & 0_{1\times 3} & \left[0\;0\;1\right] & 0_{1\times 3} & 0_{1\times 3} \end{array}\right] \end{array} \end{array}$$

In practical applications, the filter stability can be improved by tuning the parameters in the Extended Kalman Filter; the parameters include the state estimation covariance matrix P, the process noise covariance matrix Q, and the measurement noise covariance matrix R. In this study, P is a diagonal 15×15 matrix with elements

(13) $$\begin{array}{c} \begin{array}{c} P=\mathrm{diag}(\left[0_{3\times 3},0_{3\times 3},0_{3\times 3},{\left(0.1\right)}^{2}I_{3\times 3},{\left(0.1\right)}^{2}I_{3\times 3}\right] \end{array} \end{array}$$

and Q is a diagonal 12×12 matrix with elements

(14) $$\begin{array}{c} \begin{array}{c} Q=\mathrm{diag}[0_{3\times 3},0_{3\times 3},\left(1\times 10^{4}\right)I_{3\times 3},\left(1\times 10^{4}\right)I_{3\times 3}]. \end{array} \end{array}$$

R is a diagonal 4×4 matrix with elements $R={\left(0.01\;m/s^{2}\right)}^{2}I_{4\times 4}$. After tuning the Kalman Filter parameters, stable experimental results can be obtained for the pedestrian navigation algorithm.

## 4. Experiments

This section reports on the experiments designed to empirically validate the algorithm proposed in this paper. Specifically, we explore the effectiveness of the adaptive window gait frequency-based zero-velocity detection algorithm proposed in this study. In addition, we report on the experiments conducted to assess the impact of various situational factors on a pedestrian’s ability to localize and how the algorithm improves localization accuracy.

### 4.1. Settings

**Figure 7:**
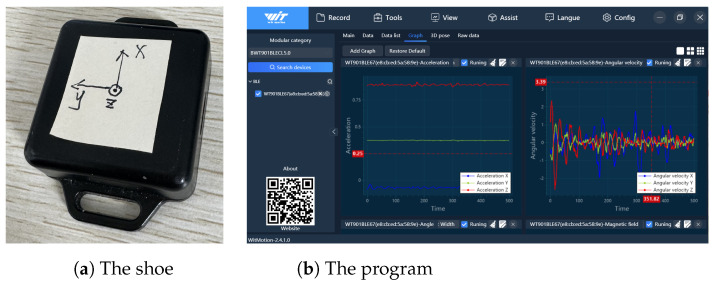
The devices used in the experiments.

As shown in Figure 7a, the inertial measurement unit has a size of 51 mm × 36 mm × 15 mm, a weight of 20 g, and integrates the ICM-42607 inertial sensor from TDK (Tokyo, Japan), which contains a three-axis accelerometer, a three-axis gyroscope, and a three-axis magnetic sensor inside. The accelerometer range is ±16 g, the gyroscope range is $\pm 2000^{\circ }$/s, and the sampling rate is set to 200 Hz. This module communicates with a laptop via Bluetooth, facilitating the transfer of data to the laptop for subsequent computation. The data collecting program, as shown in Figure 7b, was ran on a laptop.

**Figure 8:**
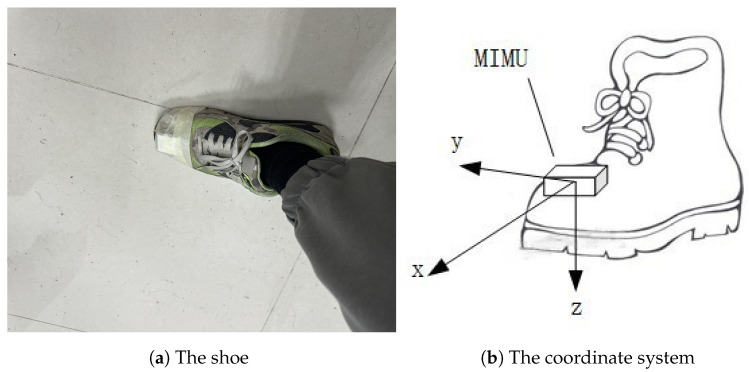
The navigation shoe.

Figure 8 illustrates the IMU-based inertial navigation shoe, where the IMU was affixed at the tip of the left foot. As shown in Figure 8b, the origin of the coordinate system coincides with the center of mass of the carrier. The *x*-axis is aligned with the longitudinal axis of the shoe, pointing forward; the *y*-axis is oriented along the lateral axis, pointing to the right; and the *z*-axis is oriented downwards, along the vertical direction. The *x*-axis, *y*-axis, and *z*-axis together form a right-handed orthogonal coordinate system.

### 4.2. Zero-Velocity Interval Detection Experiment

**Figure 9:**
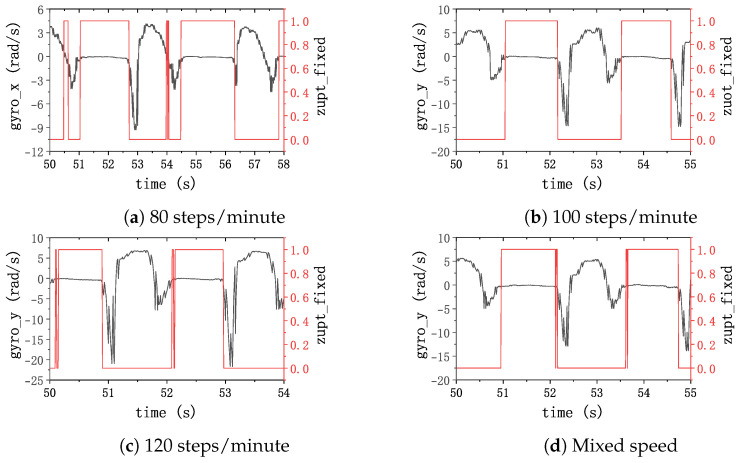
Zero-velocity detection results with a fixed threshold (100 steps/min baseline). The black lines represent the gyroscope data of the *y*-axis; the red lines represent the results of the ZVI detector. The threshold of the ZVI detector is set based on 100 steps/min.

When the subjects walked at slow, normal, fast, and randomly varying speeds, IMU data were collected. Subsequently, a fixed-threshold zero-velocity detection method was employed to identify the zero-velocity intervals (ZVI). Figure 9 illustrates the outcomes of the zero-velocity interval detection using the fixed-threshold approach alongside the output of the *y*-axis gyroscope. In this depiction, a value of 1 is assigned when the algorithm detects a zero-velocity interval and 0 otherwise. The results reveal that mismatched threshold values with the current gait frequency result in intermittent detection or undersized interval delineation, highlighting the importance of precise threshold selection for accurate zero-velocity interval detection in gait analysis and related research endeavors.

**Figure 10:**
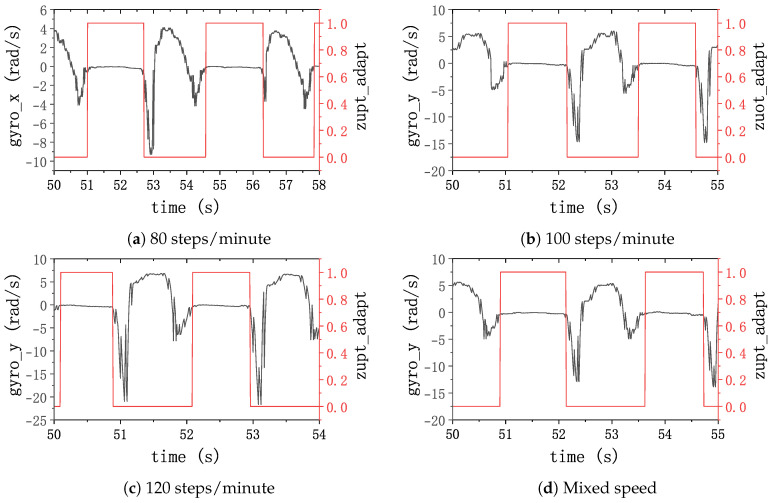
Zero-velocity detection results with an adaptive threshold. The black lines represent the gyroscope data of the *y*-axis; the red lines represent the results of the ZVI detector.

Simultaneously, a zero-velocity detection was performed using the adaptive window gait frequency-based zero-velocity detection method proposed in this study, and the results are presented in Figure 10. This illustrates that the method is robust in accurately detecting the zero-velocity interval in the pedestrian’s gait in all cases. This underscores the efficacy of the proposed method in accurately detecting zero-velocity intervals in diverse gait scenarios.

To further compare the performance of our method with others, the subjects walked along the trails at speeds of 80 steps/min, 100 steps/min, 120 steps/min, and at a mixed walking pace. Subsequently, we extracted 100 gait cycles during stable walking from each condition. Table 2 presents the detection results of the two methods under these various conditions. In this context, the fixed-threshold method employs a threshold based on a reference rate of 100 steps/min.

Table 2 illustrates that when the threshold does not match the preset reference walking speed, this method is prone to false detection. However, the zero-velocity interval detection method proposed in this paper is capable of adapting to various situations.

### 4.3. Single-Step Detection Experiments

To further investigate the impact of excessively long or short detection times during zero-velocity detection on the localization results, we conducted an analysis of the single-step solution performance during pedestrian motion. Initially, to mitigate the gait frequency fluctuations arising at the onset and conclusion of movement, we validated our approach using the average step length during walking. Participants were instructed to walk along a straight line for 10 steps (a gait cycle contains two steps) at a specified walking speed. Subsequently, the total walking distance was measured to acquire information on the participant’s step length under various gait conditions. This procedure aimed to provide insights into the performance of the single-step solution method under different speeds.

**Figure 11:**
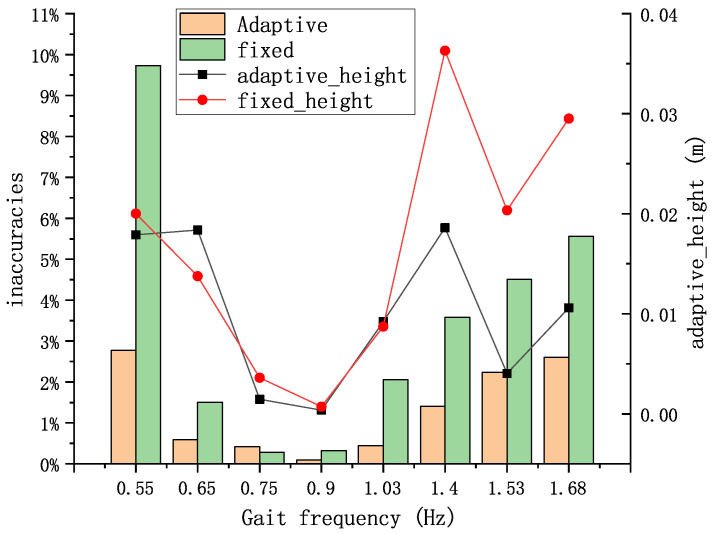
Results of the single-step error. The bar graph represents the average horizontal error rate for a single step, while the dotted line graph shows the average error value for a single step.

By employing the ZUPT-aided adaptive- and fixed-threshold methods, the trajectories and walking distances of the pedestrians were computed. This allowed for the derivation of the algorithmically estimated step length. Consequently, the compensation detection error rates under different gait frequencies were obtained, as shown in Figure 11.

**Figure 12:**
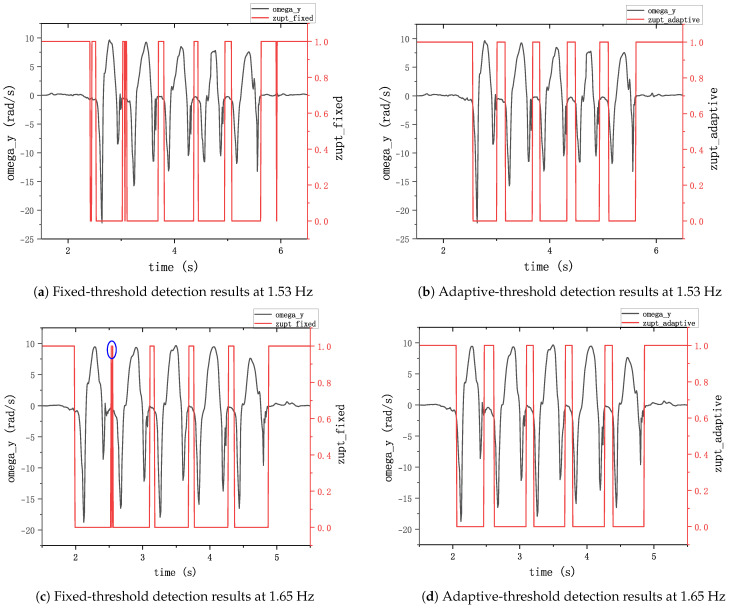
Zero-velocity detection results with an adaptive threshold. The black lines represent the gyroscope data of the *y*-axis; the red lines represent the results of the ZVI detector.

Under conditions of extremely slow walking (0.55 Hz), extremely fast walking (1.4 Hz), and running (1.53 Hz and 1.68 Hz), both algorithms exhibited high horizontal errors in step length estimation, surpassing 1%. Figure 12 demonstrates the results of the two algorithms for zero-speed interval detection in the running condition. During running, the duration of the zero-velocity interval is so small that the detection algorithm will most likely fail to detect the zero-velocity interval, and the fixed-threshold method performs demonstrable worse in the figure, failing to identify the zero-velocity interval almost completely. This has a large impact on the localization accuracy. The ZUPT algorithm is unable to correct the speed error in the zero-speed interval, which leads to an overly long single-step estimation, and this error accumulates rapidly over time, leading to poor localization accuracy. However, the performance of the adaptive-threshold method remained superior to that of the fixed-threshold algorithm. Particularly during extremely slow walking, the fixed-threshold method demonstrated a horizontal error rate approaching 10%. When pedestrians walked at greater speeds or running, the error of the height solved by the algorithm proposed in this paper was also smaller than that of the fixed-threshold method, indicating its inability to adapt to this particular gait condition.

### 4.4. Pedestrian Walking Localization Experiments

**Figure 13:**
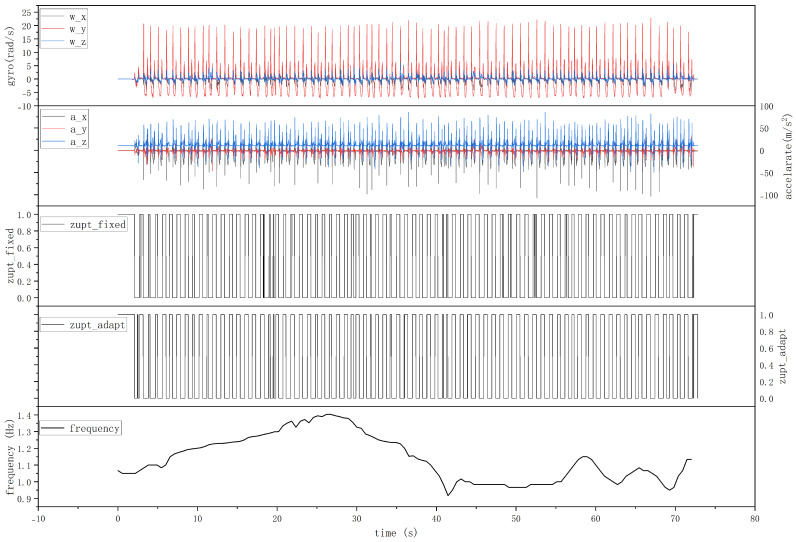
The IMU outputs, ZVI detection results, and gait frequency of the participants during walking.

This section validates the impact of the zero-velocity detection algorithm proposed in this article on the positioning accuracy within the navigation system. Here, participants were instructed to walk along a predetermined rectangular trajectory with their walking speed varying in the following sequence: slow–normal–fast–normal–slow. The total length of the trajectory was approximately 80 m. The output of the inertial sensors fixed to the subject’s shoe whilst walking is presented in the first panel of Figure 13. The third and fourth panels in Figure 13 illustrate the zero-velocity interval detection results obtained using the conventional fixed- and adaptive-threshold zero-velocity interval detection algorithm. The fifth panel in Figure 13 presents the variation in gait frequency observed during the walking experiment performed by the participants.

The results indicate that the time–frequency analysis method proposed in this paper demonstrates a remarkable capability to effectively extract information about the gait frequency during walking. This allows the algorithm to adaptively adjust various parameters based on gait frequency changes, thus improving the accuracy of the detection method. Consequently, the integration of these insights into the algorithmic framework ensures a more accurate and reliable pedestrian navigation system.

**Figure 14:**
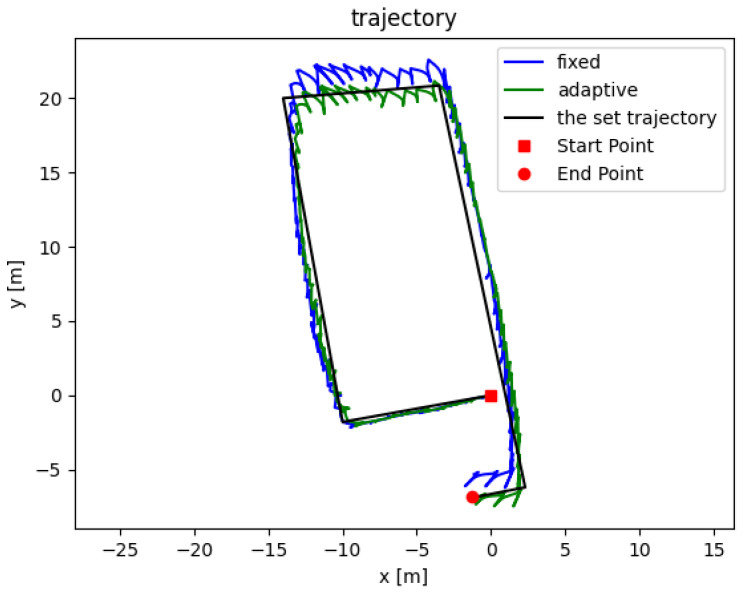
Results of walking along the rectanglar trajectory. The blue and green lines represent the trajectory calculated using the ZUPT-aided pedestrian navigation algorithm with the traditional fixed-threshold method and the adaptive ZVI detection algorithm, respectively. The black line is the set trajectory.

The inertial navigation algorithm, refined with zero-velocity correction, was subsequently utilized for calculations, and its results are illustrated in Figure 14. The black line delineates the predefined trajectory. The trajectories calculated using the ZUPT-aided fixed- and adaptive-threshold ZVI detection algorithms are represented by the blue and green lines, respectively. The trajectory marked by the blue solid line reveals a noticeable computational discrepancy in the north–south direction, extending beyond 1 m. In contrast, the trajectory calculated using the adaptive algorithm is more accurate, with a horizontal error of only 0.61 m at the endpoint. The formula for calculating the positioning error, as defined in this paper, is presented as follows:

(15) $$\begin{array}{c} \begin{array}{c} \sigma =\frac{∥\hat{p}-p_{0}∥}{s} \end{array} \end{array}$$

where $\hat{p}$ is the calculated endpoint coordinates, $p_{0}$ is the actual endpoint coordinates of the set trajectory, and *s* is the total length of the set trajectory. The experiments were repeated three times, and then we have taken the average value of the endpoint horizontal error. The navigation positioning error rate using the proposed adaptive-threshold zero-velocity detection algorithm is approximately 0.82%, while the navigation positioning error rate using the fixed-threshold zero-velocity detection method is approximately 1.13%. Based on this, the localization experiments of pedestrian walking under the four gait conditions described in Section 4.2 were carried out, and the results are shown in Table 3.

The algorithm proposed in this paper performs well under many different gaits, whereas the fixed-threshold method was insufficiently adaptable to changes in gait.

## 5. Conclusions

This paper introduces an adaptive zero-velocity detection algorithm based on the AWGF-ZVD method. Contrary to data-driven approaches, this method requires fewer datasets and computational resources. The algorithm adaptively adjusts the window parameters of the time–frequency analysis in response to variations in gait frequency during walking, thereby enhancing the algorithm’s adaptability to variations in gait frequency. Leveraging the relationship between pedestrian gait frequency and the optimal threshold, the algorithm adaptively modifies the threshold for zero-velocity detection, improving the accuracy of detecting zero-velocity intervals. Of note, excessive or extended detection can lead to a decrease in localization accuracy. The experimental results show that, compared to traditional methods, the algorithm proposed in this paper is more reliable in different pedestrian gait conditions. The algorithm proposed in this paper also performs better in situations such as slow walking and running, reflecting its adaptability to the movement state of pedestrians.

In the future study, we will pay close attention to the problem of smoothing the trajectory, height correction, and localization at more extreme slow speeds and running. By applying the AWGF-ZVD algorithm to the ZUPT algorithm, the algorithm can be adapted to the user’s different movement states.

## Figures and Tables

**Table 1:** The fitting models.

Models	Linear	Quadratic	Cubic
Mathematical Equation	y=ax+b	$y=ax^{2}+bx+c$	$y=ax^{3}+bx^{2}+cx+d$
R-Squared	0.93812	0.98139	0.9814
RMSE	8157.07	5476.63	4899.75

**Table 2:** The results of the ZVI-detector based on fixed vs. adaptive thresholds during walking.

Speed (steps/min)	Actual ZVI Number	Detected ZVI Numbers	
		Fixed-Threshold ^1^	Adaptive-Threshold
80	100	121	100
100	100	100	100
120	100	105	100
Mixed	100	124	100

^1^ The threshold of the fixed-threshold detector is based on 100 steps/min.

**Table 3:** The navigation positioning error rate using the fixed-threshold method vs. the adaptive-threshold method during walking.

Gait Frequency (Hz)	Adaptive-Threshold	Fixed-Threshold
0.67	0.86%	1.26%
0.83	0.76%	0.75%
1	0.89%	1.34%
Mixed ^1^	0.82%	1.13%

^1^ The participants walked in a slow–normal–fast–normal–slow sequence.

## Data Availability

Data are contained within the article.

## Abbreviations

The following abbreviations are used in this manuscript:

IMU	inertial measurement unit
ZUPT	zero-velocity update
GNSS	Global Navigation Satellite System
PNS	pedestrian navigation systems
AWGF-ZVD	adaptive window gait frequency-based zero-velocity detector
SINS	strapdown inertial navigation algorithm
